# The Clinical Profile of Heatstroke Patients Admitted to the Intensive Care Unit: A Case Series From a Tertiary Care Center in New Delhi, India

**DOI:** 10.7759/cureus.72114

**Published:** 2024-10-22

**Authors:** Amit Aggarwal, Rishabh Rawat, Bhupendra Sharma, Kunal Saini, Achyuth Damera, Lakshya Yadav, Siddhartha K Das

**Affiliations:** 1 Internal Medicine, Atal Bihari Vajpayee Institute of Medical Sciences & Dr. Ram Manohar Lohia Hospital, Delhi, IND

**Keywords:** exertional heat illness, heat-emergency syndromes, heat exhaustion, heat-related illnesses, heatstroke, heat syncope

## Abstract

Heat-related illnesses include a spectrum of disorders ranging from heat syncope, muscle cramps, and heat exhaustion to heat emergencies such as heatstroke. Severe heatwaves can lead to extremely high environmental temperatures and a spurt of cases of heat-related illnesses and heatstroke. The incidence of such heat-related medical emergencies was much higher in 2024 compared to previous years throughout North and Central India. Here, we are describing a case series of five patients admitted to the intensive care unit of a tertiary care center in New Delhi during the month of June 2024.

## Introduction

Heatstroke is a life-threatening condition characterized by a core body temperature above 40°C (104°F) and central nervous system dysfunction, including delirium, seizures, or coma. Prompt medical intervention is crucial to mitigate morbidity and mortality. The incidence of heatstroke is rising globally due to climate change, urbanization, and frequent heatwaves, yet it remains under-reported. Rapid recognition and aggressive treatment, such as cold-water immersion, are essential for improving outcomes [1]. Complications include acute kidney injury, rhabdomyolysis, and disseminated intravascular coagulation [2,3]. Long-term neurological deficits necessitate comprehensive follow-up care.

This case series describes five heatstroke cases admitted to the intensive care unit (ICU) of a tertiary care center in New Delhi during the peak summer of 2024, illustrating diverse presentations, complications, and outcomes. Our analysis aims to enhance the understanding of heatstroke pathophysiology, highlighting effective treatments, and preventive strategies. Various other studies from India highlight the high incidence of heat-related illnesses and increased mortality during extreme heatwaves, emphasizing the need for heightened vigilance and improved clinical care [4,5]. This series underscores the importance of vigilance among healthcare providers, especially in regions prone to extreme heat-related events.

## Case presentation

Case 1

A 38-year-old male salesman was brought to the medical emergency with a high body temperature for 10 hours and altered sensorium for eight hours. He had no known comorbidities. On arrival, his temperature was 108°F, blood pressure (BP) was 66/40 mmHg, pulse rate was 140/minute, and Glasgow Coma Scale (GCS) score was E1V1M1. Due to his low GCS, he was intubated and initiated with cold sponging and cold intravenous (IV) fluids. Neurological examination showed generalized rigidity, normal deep tendon reflexes, and bilateral sluggish reactive pupils. Laboratory results indicated acute respiratory alkalosis, thrombocytopenia, elevated liver enzymes, mild renal impairment, hyponatremia, and hyperkalemia (Table 1). Fundus examination of both eyes, electrocardiogram (ECG), chest radiograph, echocardiogram, and non-contrast CT (NCCT) of the head were unremarkable. An abdominal ultrasound showed acalculous cholecystitis. Additional tests including urine toxicology, malaria/dengue serological tests, cerebrospinal fluid (CSF) cytology, and blood cultures were negative.

**Table 1:** Laboratory investigation results of the five heatstroke patients at the time of admission to the intensive care unit, including hematological, biochemical, and inflammatory markers.

Investigation	Case 1	Case 2	Case 3	Case 4	Case 5	Normal reference range
Hemoglobin (g/dL)	14.1	16.5	12	13	12.9	13.5–17.5 (male)
Total leukocyte count (/mm^3^)	10,700	27,400	9,000	20,000	9,800	4,000–11,000
Platelet count (/mm^3^)	60,000	83,000	90,000	140,000	60,000	150,000–450,000
Total bilirubin (mg/dL)	1.6	1.0	1.0	2.4	6.2	0.3–1.2
Direct bilirubin (mg/dL)	0.4	0.5	0.4	0.64	3.36	0.1–0.3
Aspartate aminotransferase (U/L)	316	160	325	210	805	5–40
Alanine aminotransferase (U/L)	163	152	233	89	895	5–56
Alkaline phosphatase (U/L)	102	55	82	81	209	44–147
International normalized ratio	1.1	3.2	1.2	1.2	2.5	0.8–1.2
Urea (mg/dL)	26	32	50	34	87	7–20
Creatinine (mg/dL)	1.5	1.28	1.43	1.63	2.86	0.6–1.2
Sodium (mEq/L)	122	124	125	119	121	135–145
Potassium (mEq/L)	5.7	2.79	2.89	2.42	4.5	3.5–5.0
Serum magnesium (mEq/L)	1.5	1.6	1.9	1.4	1.4	1.7–2.2
Total protein (g/dL)	7.3	6.9	5.5	6.36	6.56	6.0–8.3
Serum albumin (g/dL)	4.5	3.9	2.74	3.86	3.53	3.5–5.0
Creatine kinase (U/L)	832	31039	437	3964	10012	30–200
Creatine kinase-MB (U/L)	53	66	27	54	54	<25
Lactate dehydrogenase (U/L)	317	3686	230	573	1603	140–280
C-reactive protein (mg/dL)	5.6	7.9	5.3	33	2.1	<3.0
Procalcitonin	Negative	Positive	Negative	Negative	Negative	Negative

The patient was admitted to the ICU and was treated with aggressive external cooling and cold IV fluids. Over the next two days, his sensorium improved and there were no further temperature spikes. He was extubated on day four and early physiotherapy and rehabilitation were started. The patient showed steady improvement in motor response, renal function, liver enzymes, and platelet counts. However, he continued to have residual neurological abnormalities, such as agitated behavior and hallucinations, and was not able to vocalize, though he was able to follow simple verbal commands. He was discharged subsequently with a plan for close follow-up. On outpatient department (OPD) follow-up after one month, the deficit in spoken speech was persistent but the behavioral changes and hallucinations had resolved.

Case 2

**Figure 1:**
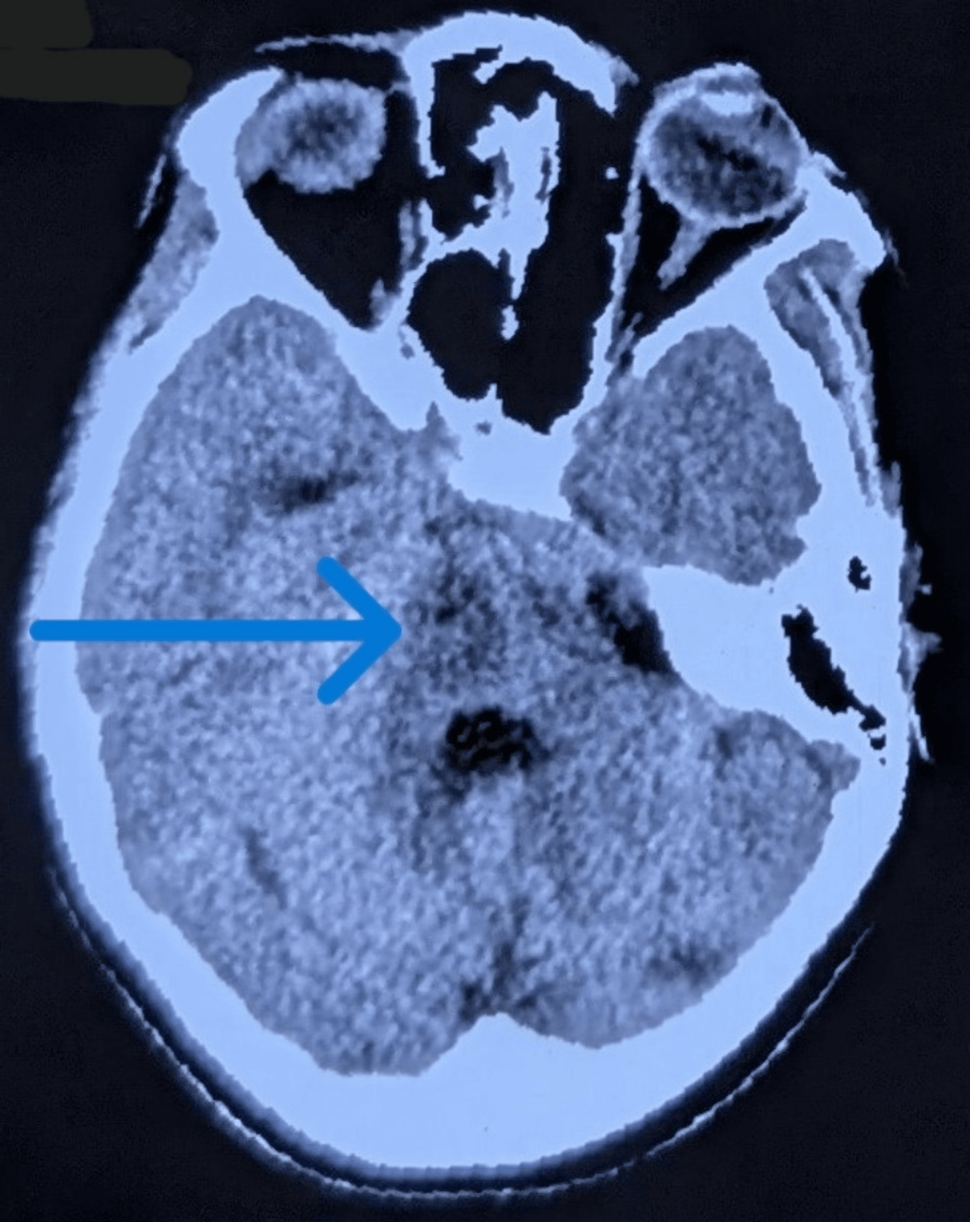
Non-contrast CT of the head showing an infarct in the right paramedian region of the pons. The blue arrow indicates the right pontine infarct.

A 54-year-old male laborer from Delhi, with a history of chronic smoking and occasional alcohol use, presented to the hospital with a one-day history of increased body temperature and altered sensorium. He had no known past illnesses. His temperature was recorded to be 106°F, BP was 200/120 mmHg, pulse rate 140/minute, respiratory rate was 35 breaths/minute, and GCS score was E1V1M4. He was intubated due to respiratory distress and poor sensorium. His pupils were dilated and reactive to light. Respiratory examination revealed bilateral infra-scapular crepitations and expiratory wheeze. He was treated with cold saline and IV antibiotics for aspiration pneumonitis. Initial investigations showed hypoxemia, metabolic acidosis, elevated total white blood cell count, thrombocytopenia, transaminitis, hypokalemia, and hyponatremia (Table 1). NCCT of the head showed an infarct in the right paramedian region of pons (Figure 1). Renal function tests and ECG were within normal limits.

The patient was shifted to the ICU. On day two of admission, the patient went into shock, requiring dual inotropes to maintain mean arterial pressure. The Sequential Organ Failure Assessment (SOFA) score was calculated to be 15. Repeat investigations indicated worsening thrombocytopenia, acute kidney injury, and elevated muscle enzymes. His coagulation profile was grossly deranged. Despite external cooling, electrolyte correction, aggressive fluid management, and supportive treatment, his condition deteriorated further. Antibiotics were escalated as his procalcitonin was also positive. Over the next two days, the patient’s shock worsened, renal function declined, and muscle enzymes and inflammatory markers showed a rising trend. Despite escalating care, including upgrading antibiotics, steroids, and maximum inotropic support, the patient did not improve. He developed cardiac arrest and succumbed on the third day.

Case 3

A 50-year-old male laborer was found unconscious at his workplace and was brought to the emergency with altered sensorium and high body temperature for two hours. He had no comorbid conditions. His temperature was recorded to be 106°F, BP was 120/70 mmHg, pulse rate was 86/minute, and GCS score was E2V1M4. Neurological examination revealed sluggishly reacting pupils. Respiratory examination revealed bilateral conducted sounds in infra-axillary areas. The patient was intubated due to poor sensorium and shifted to the ICU.

Initial investigations revealed hypoxemia, metabolic acidosis, thrombocytopenia, elevated liver enzymes, hypokalemia, and hyponatremia (Table 1). Renal function tests, ECG, and NCCT of the head were normal. Chest radiography showed right perihilar infiltration. He was managed with cold sponging, cold IV fluids, and IV antibiotics. As his sensorium improved, he was extubated on day four of the ICU stay. By day six, thrombocytopenia, transaminitis, and electrolyte imbalances had resolved. His clinical condition improved but there was a residual deficit of decreased verbal output and impaired comprehension of spoken words. He was discharged with a plan for close follow-up. On OPD follow-up after one month, the comprehension of spoken words had partly recovered but decreased verbal output persisted.

Case 4

**Figure 2:**
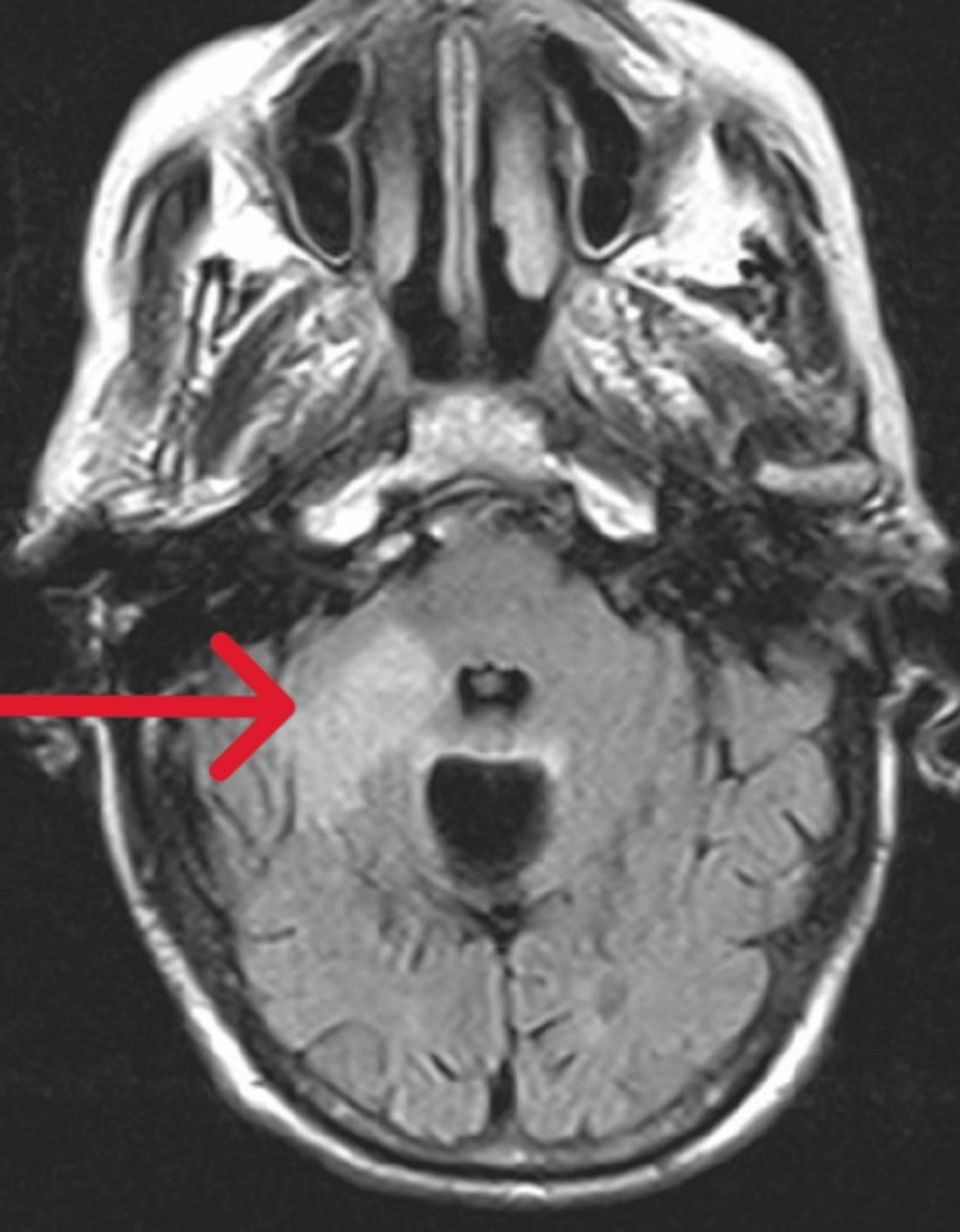
Fluid-attenuated inversion recovery axial MRI showing a right pontine infarct. The red arrow points to the area of infarction in the right pons.

A 60-year-old hypertensive male presented to the medical emergency with sudden-onset altered sensorium and a temperature of 108°F. On examination, his pulse rate was 146/minute, BP was 100/60 mmHg, and GCS score was E1V1M1. Neurological examination showed normal deep tendon reflexes, flexor plantar responses, and reactive pupils bilaterally. Respiratory examination revealed inspiratory crepitations in the right mammary and infra-axillary region. He was intubated due to poor sensorium and managed with cold sponging, IV fluids, and broad-spectrum IV antibiotics. Initial investigations indicated metabolic acidosis, leukocytosis, thrombocytopenia, elevated liver enzymes, hyponatremia, and hypokalemia (Table 1). Blood glucose levels, renal function tests, fundus examination, and echocardiography were normal. MRI of the brain showed a right pontine infarct (Figure 2).

The patient was shifted to ICU, where aggressive cooling and supportive care were continued. Over the next three days, the patient’s sensorium gradually improved, and he was eventually extubated on day four of the ICU stay. He showed steady improvement in speech and motor responses, and early physiotherapy and rehabilitation were initiated. The patient was discharged from the ICU without any residual neurological deficit. On OPD follow-up after one month, the patient was healthy.

Case 5

A 67-year-old male presented to the medical emergency with sudden-onset altered sensorium for three hours with no verbal output and no response to any stimuli. The patient had a history of schizophrenia and not taking any medications. He was a chronic bidi smoker. On examination, his pulse rate was 146/minute, BP was 140/80 mmHg, and core temperature was 108°F. He had a GCS score of E1V1M1. Neurological examination showed increased tone in all four limbs and sluggishly reactive pupils. He was intubated due to poor sensorium and was shifted to the ICU where he was managed with cold sponging, cold IV fluids, and broad-spectrum IV antibiotics.

Initial investigations showed metabolic acidosis, leukocytosis, elevated liver enzymes, hyponatremia, and renal impairment (Table 1). Blood glucose, fundoscopy, ECG, echocardiography, NCCT of the head, urine R/M, and toxicology screen revealed no abnormality. He had raised creatine kinase, creatine kinase-MB, D-dimer, and C-reactive protein levels. The patient went into shock requiring dual inotropic support. He developed multiorgan dysfunction syndrome, with worsening leukocytosis, renal function, thrombocytopenia, and coagulopathy. After five days of ICU stay, his clinical condition gradually improved and inotropic support was tapered. However, sensorium, motor response, and respiratory efforts did not improve. On day 12 of ICU admission, he was tracheostomized and put on T-piece ventilation. After 20 days of ICU stay, his GCS score was E4VTM3 at which point he was discharged with a plan to follow up closely. On serial OPD follow-up visits, the tracheostomy tube was downsized and removed. After one month, the patient had normal spontaneous respiratory efforts but there was still a residual deficit in motor response, comprehension of spoken words, and verbal output.

## Discussion

This case series illustrates the diverse clinical presentations, complications, and management of heatstroke during the intense heatwave between June 15th and 20th, 2024 in New Delhi. Heatstroke, resulting from thermoregulation failure, can be classified into classic and exertional types. Classic heatstroke affects vulnerable populations during heatwaves, while exertional heatstroke occurs in healthy individuals engaging in strenuous activities in hot environments [6,7]. The pathophysiology involves excessive heat production and impaired heat dissipation, leading to tissue injury and a systemic inflammatory response that can result in multiorgan failure [8].

The five cases in this series highlight the variability in heatstroke presentations. Cases ranged from a young male with multiorgan involvement who recovered but had persistent speech issues (Case 1) to a middle-aged laborer with multisystem failure who could not be revived (Case 2). Another laborer developed heatstroke at his workplace but improved with treatment, underscoring the need for preventive measures in high-risk professions (Case 3). The final two cases involved elderly males with classic heatstroke and multisystem involvement, both showing improvement, although one had persistent speech and motor deficits (Cases 4 and 5).

Clinical evidence

An Indian study in 2012 reported 13 out of 26 heatstroke patients with hypokalemia and all with elevated creatinine phosphokinase levels. MRI scans showed diffusion restrictions and edema in multiple brain regions in five patients. Sixteen required mechanical ventilation and six needed vasopressors despite fluid resuscitation [9]. Another study found that tachycardia, hypotension, low GCS scores, high leukocyte count, and low bicarbonate were mortality indicators in 84 heatstroke patients [10]. In an observational study among 455 patients, the most common symptoms experienced were increased temperature, muscle weakness, and dehydration [11]. In another study on 27 heatstroke cases, 66% had a SOFA score >2, with central nervous system dysfunction and respiratory distress being prevalent [12].

Management and preventive strategies

Effective and immediate cooling is crucial in treating heatstroke to prevent mortality and long-term complications. Methods include ice water immersion, evaporative cooling, and ice packs, with ice water immersion being the most effective but logistically challenging [13]. Maintaining the airway, breathing, circulation, and continuous monitoring of vital signs, core temperature, cardiac rhythm, and urine output is essential. Frequent reassessment and managing complications are key, along with long-term neurological follow-up for cognitive and focal deficits.

Heatstroke can lead to complications such as acute kidney injury, liver dysfunction, coagulopathy, and neurological impairment [6]. Significant laboratory abnormalities included elevated liver enzymes, renal impairment, and metabolic disturbances. Rhabdomyolysis in Case 2 required aggressive fluid resuscitation to prevent renal failure [14]. Neurological outcomes varied. Case 1 showed agitated behavior and hallucinations but could follow commands; Case 3 had decreased verbal output and impaired comprehension; and Case 5 experienced delayed recovery in spontaneous respiratory efforts and residual verbal and motor deficits.

Preventing heatstroke involves public health interventions such as cooling centers and community education during heatwaves. Ensuring adequate hydration, rest breaks, and cooling measures can significantly reduce incidence [15]. Long-term follow-up of survivors is necessary to manage chronic complications such as cognitive impairment and renal dysfunction [16]. Regular monitoring and supportive care are essential to improve the quality of life for survivors.

## Conclusions

This series is unique in its demonstration of the broad spectrum of heatstroke manifestations and outcomes, emphasizing the importance of tailored management strategies for different patient profiles. This case series is limited by its small sample size and single-center design. Future research should focus on larger, multicenter studies to validate these findings and explore optimal management strategies for heatstroke. Investigating the role of novel cooling techniques, pharmacological interventions, and long-term outcomes will provide further insights into improving patient care. Additionally, developing comprehensive public health policies and workplace regulations can help prevent heat stroke and mitigate its impact on vulnerable populations.

## References

[1] Rublee C, Dresser C, Giudice C, Lemery J, Sorensen C (2021). Evidence-based heatstroke management in the emergency department. West J Emerg Med.

[2] Jung YS, Kim HH, Yang HW, Choi S (2020). Targeted temperature management in patients with severe heatstroke: three case reports and treatment recommendations. Medicine (Baltimore).

[3] Asmara IG (2020). Diagnosis and management of heatstroke. Acta Med Indones.

[4] Saha MV, Davis RE, Hondula DM (2014). Mortality displacement as a function of heat event strength in 7 US cities. Am J Epidemiol.

[5] Azhar GS, Mavalankar D, Nori-Sarma A (2014). Heat-related mortality in India: excess all-cause mortality associated with the 2010 Ahmedabad heat wave. PLoS One.

[6] Bouchama A, Knochel JP (2002). Heat stroke. N Engl J Med.

[7] Leon LR, Bouchama A (2015). Heat stroke. Compr Physiol.

[8] Epstein Y, Yanovich R (2019). Heatstroke. N Engl J Med.

[9] Kalaiselvan MS, Renuka MK, Arunkumar AS (2015). A retrospective study of clinical profile and outcomes of critically ill patients with heat-related illness. Indian J Anaesth.

[10] Roy DB, Khatri HV, Parikh AP (2019). Study of epidemiological determinants of patients presented with “heat wave related illness” admitted in tertiary care center. Natl J Community Med.

[11] Nagaonkar S, Ukarande BV, Wadagale AV (2019). A study of clinic socio demographic profile of the patients admitted and referred with heat related illnesses in last three years at tertiary health care centre of Latur, Maharashtra. MedPulse Int J Community Med.

[12] Ninan GA, Gunasekaran K, Jayakaran JA, Johnson J, Abhilash K, Pichamuthu K, Iyadurai R (2020). Heat-related illness-clinical profile and predictors of outcome from a healthcare center in South India. J Family Med Prim Care.

[13] Casa DJ, DeMartini JK, Bergeron MF (2015). National Athletic Trainers' Association Position Statement: exertional heat illnesses. J Athl Train.

[14] Bosch X, Poch E, Grau JM (2009). Rhabdomyolysis and acute kidney injury. N Engl J Med.

[15] McDermott BP, Casa DJ, Ganio MS, Lopez RM, Yeargin SW, Armstrong LE, Maresh CM (2009). Acute whole-body cooling for exercise-induced hyperthermia: a systematic review. J Athl Train.

[16] Bouchama A, Dehbi M, Chaves-Carballo E (2007). Cooling and hemodynamic management in heatstroke: practical recommendations. Crit Care.

